# Why Turing’s Computable Numbers Are Only Non-Constructively Closed Under Addition

**DOI:** 10.3390/e28010071

**Published:** 2026-01-07

**Authors:** Jeff Edmonds

**Affiliations:** Department of Electrical Engineering and Computer Science, York University, Toronto, ON M3J 1P3, Canada; jeff@yorku.ca

**Keywords:** Kolmogorov complexity, Turing-computable, digit-by-digit, carry propagation, *ϵ*-approximations

## Abstract

Kolmogorov complexity asks whether a string can be outputted by a Turing Machine (TM) whose description is shorter. Analogously, a real number is considered computable if a Turing machine can generate its decimal expansion. The modern ϵ-approximation definition of computability, widely used in practical computation, ensures that computable reals are constructively closed under addition. However, Turing’s original 1936 digit-by-digit notion, which demands the direct output of the n-th digit, presents a stark divergence. Though the set of Turing-computable reals is not constructively closed under addition, we prove that a Turing machine capable of computing x+y non-constructively exists. The core constructive computational barrier arises from determining the ones digit of a sum like $0.33\bar{3}+0.66\bar{6}=0.99\bar{9}=1.00\bar{0}$. This particular example is ambiguous because both $0.99\bar{9}$ and $1.00\bar{0}$ are legitimate decimal representations of the same number. However, if any of the infinite number of 3s in the first term is changed to a 2 (e.g., $0.33\ldots 32\ldots +0.66\bar{6}$), the sum’s leading digit is definitely zero. Conversely, if it is changed to a 4 (e.g., $0.33\ldots 34\ldots +0.66\bar{6}$), the leading digit is definitely one. This implies an inherent undecidability in determining these digits. Recent papers and our work address this issue. Hamkins provides an informal argument, while Berthelette et al. present more complicated formal proof, and our contribution offers a simple reduction to the Halting Problem. We demonstrate that determining when carry propagation stops can be resolved with a single query to an oracle that tells if and when a given TM halts. Because a concrete answer to this query exists, so does a TM computing the digits of x+y, though the proof is non-constructive. As far as we know, the analogous question for multiplication remains open. This, we feel, is an interesting addition to the story. This reveals a subtle but significant difference between the modern ϵ-approximation definition and Turing’s original 1936 digit-by-digit notion of a computable number, as well as between constructive and non-constructive proof. This issue of computability and numerical precision ties into algorithmic information and Kolmogorov complexity.

## 1. Introduction

Computer science, effectively founded by Turing, relies on the ability to compute the sum of computable numbers. While it is often taken for granted that the set of computable numbers is closed under addition, the validity of this statement depends entirely on the definition of “computability” being used. There are two distinct definitions in the literature: Turing’s original 1936 digit-by-digit definition (which we will call **Turing-computable**) and the modern definition based on approximation (which we will call **ϵ-computable**).

Under the ϵ-computable definition, real numbers are constructively closed under addition and multiplication. Modern computable analysis largely adopted this definition following the work of Pour-El and Richards [1], precisely because Turing’s original definition lacks this constructive closure.

**Turing-computable (Digit-by-Digit):** A real number *x* is Turing-computable if there exists a Turing machine $M_{x}\left(n\right)$ that, on input *n*, outputs the n-th digit of the decimal expansion of *x*. The number is said to be *weakly printable* digit-by-digit if it allows an infinite string of 9s.
**ϵ-computable (Approximation):** A real number *x* is ϵ-computable if there exists a Turing machine $M_{x}\left(\epsilon \right)$ that, on input a rational ϵ>0, outputs a rational number $x^{\prime }$ such that $|x^{\prime }-x|\leq \epsilon$.

The equivalence between these two definitions is well-known, yet subtle. While every Turing-computable number is ϵ-computable and vice versa, the transformation between these forms is not always constructive. The goal of this paper is to explore the gap between these definitions, specifically regarding the closure of addition.

We distinguish between two types of closure:

**Constructive Closure:** There exists a uniform algorithm that, given descriptions of machines for *x* and *y*, produces a machine for x+y.
**Non-Constructive Closure:** For any computable *x* and *y*, a machine for x+y exists, though no algorithm may exist to find it.

We prove the following:

**Constructive Proof:** According to **Turing-computable** definition, computable numbers are **not constructively** closed under addition or multiplication, because it is undecidable to compute the n-th digit of x+y from Turing machines $M_{x}\left(n\right)$ and $M_{y}\left(n\right)$. In fact, it is undecidable to determine whether x≥1.
**Non-Constructive Proof:** Under this same definition, the set of computable real numbers is **non-constructively** closed under addition: We can prove that a machine $M_{x+y}\left(n\right)$ computing the digits of x+y exists, even though we cannot necessarily construct it.

There are three proofs of this first result: informal arguments by Hamkins [2], a formal proof using Kleene’s Recursion Theorem by Berthelette et al. [3], and the proof presented in this paper. All three leverage the ambiguity introduced by carry propagation. However, our approach differs by offering a direct reduction to the Halting Problem without relying on Kleene’s Recursion Theorem, which we believe makes the result conceptually simpler and more accessible.

In conclusion, our work delineates a clear computational gap between approximation and digit-level computation, and between constructive and non-constructive proofs.

## 2. Previous Work

In 1912, Émile Borel [4] was perhaps the first to write explicitly about real numbers that could be “defined” or “constructed” using finite procedures, though his approach was more philosophical and intuitive than formal. He laid conceptual groundwork for what would later become formalized notions of computability. In 1936, Alan Turing, in his seminal paper *On Computable Numbers, with an Application to the Entscheidungsproblem* [5], proved that the Halting Problem is undecidable. As part of this development, he defined a real number *x* to be **computable** if there exists a Turing machine that outputs its decimal (or binary) expansion digit by digit. This laid the foundation for what we now call computable analysis. Turing was already aware of the subtleties involved in digit computation, and he implicitly recognized the difficulty posed by issues like carry propagation (e.g., in his treatment of convergence). However, he did not explicitly formalize or reduce the Halting Problem to questions of digit-wise arithmetic. The specific problem of undecidability of digit-level addition or carry propagation was not addressed in his original work. In 1949, Specker [6] constructed a computable sequence of rational numbers that converges to a non-computable real limit, showing that the computable reals are not closed under limits. This important insight into non-constructive convergence is not directly related to digit-wise arithmetic or carry propagation. In 1968, Richardson [7] proved that it is undecidable to determine whether certain symbolic expressions involving real functions (e.g., sin(x), exp(x)) are identically zero. While this result falls within real analysis and computability, it focuses on symbolic identities rather than decimal digit computation. In their landmark 1989 book, Pour-El and Richards [8] (see also Weihrauch [9]) demonstrated that the set of computable real numbers is closed under standard arithmetic operations, including addition and multiplication. However, their model of computation is based on ϵ-approximation: a number is computable if it can be approximated to arbitrary precision. This differs from Turing’s original digit-by-digit model, and their results do not establish constructive closure under that older definition. In 1991, Goldberg [10] highlighted the *table-maker’s dilemma*, which refers to the difficulty of rounding results correctly based on finite-precision computations. While this is a practical concern in numerical computing, it does not imply undecidability, nor does it concern Turing’s digit-by-digit model. In 2006, Braverman [11] showed that even basic comparisons between computable reals—such as equality and ordering—can be undecidable in general. He proved this by reducing these tasks to the Halting Problem. His work lies within the framework of computable analysis using ϵ-approximations and did not directly address digit-by-digit arithmetic or the possibility of infinite carry chains. In 2018, Joel David Hamkins, in his accessible blog post [2], argues for the result discussed in this paper: the uncomputability of addition in Turing’s digit-by-digit model. He uses examples where determining the sum’s digits (like distinguishing between $0.99\bar{9}$ and $1.00\bar{0}$) requires examining an infinite number of digits from the input numbers. He states that similar examples show that multiplication and many other very simple functions are not computable if one insists that a computable number is an algorithm enumerating the digits of the number. He also notes that Kleene’s Recursion Theorem can be used to formalize this. Kleene’s Recursion Theorem [12] (KRT) is a fundamental result in computability theory that enables the construction of self-referential programs or Turing machines. In essence, it states that for any computable operation on programs, there exists a program that, when subjected to that operation, behaves as if it has access to its own source code or index. This allows for the creation of “pathological” self-referential machines that can be used to prove various undecidability results, including the impossibility of a general algorithm for digit-by-digit addition.

In 2024, Berthelette, Brassard, and Coiteux-Roy [3] provided a more rigorous and formal treatment, largely filling out Hamkins’s arguments concerning the uncomputability of multiplication by 3 in the weakly printable model, also utilizing Kleene’s Recursion Theorem. Their work delves into the specific ambiguities arising from infinite strings of 9s (or 1s in binary) and how these relate to the inherent uncomputability of arithmetic operations under Turing’s original definition.

We believe our independent discovery and formalization of the same phenomenon for addition is conceptually simpler. Our approach directly encodes the carry propagation issue into the Halting Problem, offering a straightforward reduction that is accessible for undergraduate students. This new view also led us to proving that non-constructively there exists a TM that computes the digits of x+y.

## 3. Positive Results: Constructive and Non-Constructive Closure

For completeness, we include Pour-El and Richards’ [1] result from 1979.

**Theorem** **1** (Constructive Closure under ϵ-Approximation)**.** *Given Turing machines $M_{x}\left(\epsilon^{\prime }\right)$ and $M_{y}\left(\epsilon^{\prime }\right)$ that compute real numbers x≥0 and y≥0 to any precision $\epsilon^{\prime }$, and a target precision ϵ, one can construct a Turing machine $M_{x\times y}\left(\epsilon \right)$ that approximates x×y within ϵ.*

**Proof.**  We first call $M_{x}\left(1\right)$ and $M_{y}\left(1\right)$ to approximate *x* and *y* to within 1, allowing us to determine upper bounds ⌈x⌉ and ⌈y⌉. Set $\epsilon^{\prime }=\frac{\epsilon }{2\max(\lceil x\rceil +\lceil y\rceil ,1)}$, and compute more refined approximations $x^{\prime }=M_{x}\left(\epsilon^{\prime }\right)$ and $y^{\prime }=M_{y}\left(\epsilon^{\prime }\right)$ that are within $\epsilon^{\prime }$ of *x* and *y*. Since these approximations must have finite decimal expansions, we can multiply them exactly to obtain $z^{\prime }=x^{\prime }\times y^{\prime }$. We prove that this meets our requirement that $|z^{\prime }-xy|\leq \epsilon$:

$$|z^{\prime }-xy|\leq \left(x+\epsilon^{\prime }\right)\times \left(y+\epsilon^{\prime }\right)-xy=\left(x+y\right)\epsilon^{\prime }+\epsilon^{\prime 2}\leq 2\left(\left\lceil x\right\rceil +\left\lceil y\right\rceil \right)\epsilon^{\prime }=\epsilon$$

□

**Theorem** **2** (Non-Constructive Closure under Turing’s Digit-by-Digit Model)**.** *Given Turing machines $M_{x}\left(n^{\prime }\right)$ and $M_{y}\left(n^{\prime }\right)$ that compute the digits of x and y, we can prove that a machine $M_{x+y}\left(n\right)$ computing the digits of x+y exists.*

**Proof.**  Let $x_{n}$, $y_{n}$, and $z_{n}$ denote the *n*-th digits of *x*, *y*, and z=x+y, respectively. The key issue in computing $z_{n}$ is carry propagation:

$$z_{n}=\left(x_{n}+y_{n}+c_{n}\right)\;\mathrm{mod}\;10,$$

where $c_{n}\in \left\{0,1\right\}$ is the carry into the *n*-th position. Each digit position $n^{\prime }>n$ may

- **Create a carry** if $x_{n^{\prime }}+y_{n^{\prime }}\geq 10$;
- **Propagate a carry** if $x_{n^{\prime }}+y_{n^{\prime }}=9$;
- **Destroy a carry** if $x_{n^{\prime }}+y_{n^{\prime }}\leq 8$.

Define LastP(x,y) (a value strictly determined by *x* and *y*) as follows:

- *∞* if there are an infinite number of non-propagating digits $n^{\prime }$, i.e., those for which $x_{n^{\prime }}+y_{n^{\prime }}\neq 9$.
- Otherwise, it is defined as the largest index $n^{\prime }$ for which this is the case.

Given the value LastP(x,y) hardcoded into the machine, one can compute the n-th digit $z_{n}$ of x+y as follows: If LastP(x,y)=∞, there is always a non-propagating position after *n*, and the machine can search for it without worrying about an infinite loop. If $\mathrm{LastP}\left(x,y\right)=n_{\mathrm{last}}$, the machine need not search past $n_{\mathrm{last}}$. Knowing whether the first non-propagating position is a creator or destroyer of carry, the machine computes the carry $c_{n}$ at position *n* and hence the digit $z_{n}$. Since for every *x* and *y* the value LastP(x,y) exists, a TM $M_{x+y}\left(n\right)$ with this value hardwired exists. □

**Remark** **1.** *Note that the proof above is **non-constructive**. While the value LastP(x,y) exists, computing it is Turing-equivalent to solving the Halting Problem (as shown in the next section). Therefore, we cannot uniformly construct this machine from $M_{x}$ and $M_{y}$.*

## 4. Lower Bounds on Closure Properties

First, we formally define the Halting Problem and the associated notation.

**Definition** **1** (Halting Problem)**.** *Let Halt(M) be the predicate that is true if Turing machine M halts on empty input, and false otherwise. It is well known that determining Halt(M) is undecidable.*

**Theorem** **3** (No Constructive Closure under Turing’s Digit-by-Digit Model)**.**

- *According to **Turing-computable** definition, computable numbers are **not constructively** closed under addition, because it is undecidable to compute the n-th digit of x+y from descriptions of Turing machines $M_{x}\left(n^{\prime }\right)$ and $M_{y}\left(n^{\prime }\right)$.*
- *Similarity for multiplication.*
- *In fact, it is undecidable to determine whether a number $x_{M}$ defined by machine M satisfies $x_{M}\geq 1$.*
- *If the definitions do not require the answer to switch from the non-standard $x+y=0.\bar{9}$ to $1.\bar{0}$, then a similar proof works.*

**Proof.** 

- We show that if one could constructively compute the digit-by-digit sum x+y, then one could decide whether a given Turing machine halts, which is impossible. Let *M* be an arbitrary Turing machine. If *M* halts in *T* steps, then we will define the number $x_{M}$ as $x_{M}=0.\underset{T\;\mathrm{times}}{\underset{︸}{33\ldots 3}}00\bar{0}$. The TM that computes the digits of $x_{M}$ is **defined** as follows: Simulate *M* for *n* steps. If *M* does not halt within *n* steps, output digit 3. Otherwise, output digit 0. Let $M_{y}\left(n\right)$ always output 6. Now consider $z=x_{M}+y$:
  - If *M* halts in *T* steps, then $x_{M}+y=0.\underset{T\;\mathrm{times}}{\underset{︸}{33\ldots 3}}00\bar{0}+0.66\bar{6}=0.\underset{T\;\mathrm{times}}{\underset{︸}{99\ldots 9}}66\bar{6}<1$.
  - If *M* does not halt, then $x_{M}+y=0.33\bar{3}+0.66\bar{6}=0.99\bar{9}=1$.
  Note that $M_{x+y}\left(0\right)$, which is the ones digit of $x_{M}+y$, is 1 if and only if *M* does not halt. If we could compute this value from descriptions of Turing machines $M_{x}\left(n\right)$ and $M_{y}\left(n\right)$, then we could solve ¬Halt(M). This proves that constructive closure under addition contradicts the undecidability of the Halting Problem.
- We repeat the same argument for multiplication. Let y=3.0 (exact), so that
  - If *M* halts, $x<\frac{1}{3}\Rightarrow x\times y<1$;
  - If *M* does not halt, $x=\frac{1}{3}\Rightarrow x\times y=1$.
  Again, the leading digit of x×y tells us whether *M* halts, violating undecidability.
- It is similarly undecidable to determine whether the real value *x* represented by $M_{x}\left(n\right)$ is at least 1, shown by switching $M_{x}\left(n\right)$ to output 9s instead of 3s.
- The above proof breaks down if the definitions do not require the answer to switch from the non-standard $x+y=0.\bar{9}$ to 1.0000. However, a modified proof still works. Change $M_{x}\left(n\right)$ to output 4s instead of 0s when *M* halts, giving the following:
  - If *M* halts in *T* steps, then $x+y=0.\underset{T\;\mathrm{times}}{\underset{︸}{33\ldots 3}}44\bar{4}+0.66\bar{6}=1.\underset{T\;\mathrm{times}}{\underset{︸}{00\ldots 0}}11\bar{1}\geq 1$.
  - If *M* does not halt, then $x+y=0.33\bar{3}+0.66\bar{6}=0.99\bar{9}$.
  So, even without normalization of repeating 9s, summation remains undecidable.

□

## 5. Conclusions

While the computable reals are closed under addition and multiplication in the ϵ-approximation sense, they are not constructively closed under these operations in Turing’s digit-by-digit model. That is, although a Turing machine $M_{x+y}\left(n\right)$ computing the n-th digit of x+y can be shown to exist, it is undecidable to construct such a machine from $M_{x}\left(n\right)$ and $M_{y}\left(n\right)$. This reveals a subtle but important distinction between the two definitions of computable numbers and between constructive proofs and non-constructive existence results in computability theory.

## Data Availability

The original contributions presented in this study are included in the article. Further inquiries can be directed to the corresponding author.
